# Perceptions, Practices, and Challenges Regarding Menstrual Hygiene Among Women in Karachi, Pakistan: A Comparison Between General Population and Healthcare Workers

**DOI:** 10.7759/cureus.9894

**Published:** 2020-08-20

**Authors:** Shajeea Arshad Ali, Mariam Baloch, Lubna Riaz, Ayman Iqbal, Ramsha Riaz, Bushra Perveen, Maham Siddiqui, Asadullah Arshad Ali

**Affiliations:** 1 Internal Medicine, Dow Medical College, Dow University of Health Sciences, Karachi, PAK; 2 Forensic Medicine and Toxicology, Dow Medical College, Dow University of Health Sciences, Karachi, PAK; 3 Internal Medicine, Dow International Medical College, Dow University of Health Sciences, Karachi, PAK

**Keywords:** menstruation, menarche, menstrual cycle, menstrual management, healthcare workers, reproductive health, reproductive tract infections, pakistan, menstrual hygiene, low- and middle-income country

## Abstract

Background

Menstruation is a natural physiological phenomenon, yet considered a stigmatized subject, particularly in low- and middle-income countries like Pakistan. It is seldom discussed openly, leading to flow of incorrect and incomplete knowledge. The resultant unhealthy practices not only affect the health of the menstruator but can also contribute to considerable psychosocial stress. Menstrual hygiene management (MHM) is an important facet, which is associated with a variety of practices and beliefs in different parts of the world, some of which may not be correct. Identifying these poor methods is necessary in order to rectify them. Hence, our study aimed at determining the level of knowledge, beliefs, and practices pertaining to menstruation in the general female populace of Karachi. Healthcare workers have a potential role in the dissemination of authentic knowledge and practices. Therefore, we assessed and compared the accuracy and reliability of the aforementioned parameters in them and the need for an educational intervention.

Methods

A descriptive, cross-sectional study was conducted on females visiting the Gynecologic and Obstetrics Outpatient Department (OPD), and healthcare workers employed at Dr. Ruth K. M. Pfau Civil Hospital Karachi and Dow University Hospital Ojha. Using non-probability convenience sampling, a self-structured questionnaire was used to collect data from 353 respondents over a duration of three months from October 2019 to January 2020. Data was tabulated in Statistical Package for the Social Sciences (SPSS) version 24.0 (IBM Corp., Armonk, NY, US). In accordance with the objectives of the study, descriptive analysis was performed, and data was presented in the form of frequencies and percentages.

Results

Of the 353 participants, 176 were from the general population and 177 were healthcare workers. At menarche, only 28.4% of the general population and 29.4% of healthcare workers had an idea of menses and proper placement of absorbent. Significantly lower number of females from the general population were found to be aware of tampons and menstrual cups (15.9% and 11.4% respectively) as compared to healthcare workers. For both groups, the source of knowledge was mostly their mother. The study showed that 77.8% of the general population and 66.1% of healthcare workers avoided bathing on certain days during menses, with the most common reason being that "it causes irregular flow". As compared to healthcare workers, a significantly higher number of women from the general population had restrictions of activity (53.4%) and avoided washing of groin area during menstruation (31.2%). Majority of women from the general population mentioned that they were scared when they menstruated for the first time. The most common absorbent used by respondents was pads, followed by cloth. The data showed 64.2% of females from the general population and 28.8% of healthcare workers abstained from eating certain foods. Seeking treatment for gynecological issues was not widespread among respondents.

Conclusion

Our study demonstrated insufficient menstrual knowledge, and consequent incorrect practices in the female population of Karachi. Destigmatizing menstruation and educating women and young girls is indispensable to overcoming this gap. At the same time, reinforcing the availability of MHM products is long overdue and is a crucial milestone towards facilitation of MHM for the women of Pakistan.

## Introduction

Menstruation is an essential phenomenon transitioning a female’s body from childhood to pubertal age. It is a life-altering event where, although ceremonial attention to “coming of age” is paid, very little factual information is given regarding its management and practices [1]. According to the United Nations Children's Fund (UNICEF), menstrual hygiene management (MHM) is hygiene related to the menstrual process. MHM is defined as women and adolescent girls having access to menstrual hygiene products, soap and water, and adequate sanitation facilities throughout the duration of a menstrual cycle. It also includes the aspect of women understanding the basic facts related to the menstrual cycle and how to manage it with confidence and dignity [2].

MHM begins at menarche; albeit the concepts, beliefs, and practices employed vary in different parts of the world owing to a variety of factors including access to water, sanitation, and hygiene (WASH) facilities at the household level, socioeconomic status, education of the menstruator, source of knowledge, divergent ethnicities, and religious and cultural differences [3].

There is a stigma and taboo associated with menstruation, especially in low- and middle-income countries (LMICs). It is considered ‘dirty’ or ‘impure’, something that should be ‘shrouded in secrecy’ and suffered in silence [4]. Most of the women are hesitant to discuss matters pertaining to sexual health and do not discuss it openly, which serves as a major barrier to proper education regarding menstrual hygiene [3]. With inadequate information being disseminated, they harbor myths and preconceived notions that are reflected in their unhealthy menstrual practices. This not only has medical implications like increased risk of urinary tract infections (UTIs) and reproductive tract infections (bacterial vaginosis, candidiasis, vaginal scabies), but can also contribute to considerable psychosocial stress [5-7].

There has been a significant amount of research on myths and traditional strategies governing the menstruation in other resource-limited countries like Ghana, Tanzania, Ethiopia, Nepal, and India [3,4]. However, the same cannot be said for Pakistan, which also has straitened resources and similar MHM state. The topic has not been sufficiently studied with only a few cross-sectional studies conducted in some cities of Pakistan such as Quetta, Peshawar, and Lahore [8-10]. There is a lack of availability of the recent data in the literature regarding this topic in Karachi, which is the major metropolis of the country. The recent Census of Pakistan of the year 2017 states a figure of 101,331,000 (48.77%) females from the total population of 207,774,520 [11]. Considering that the females constitute an almost equal fraction of the population, the lack of research of their most basic life event is dismal, to say the least. Therefore, to bridge this gap, the primary objective of our investigation was to determine the knowledge, perceptions, practices, and challenges regarding menstruation faced by the women in Karachi, Pakistan.

Furthermore, the World Health Organization (WHO) defines the health workforce as “all people engaged in actions whose primary intent is to enhance health” [12]. These include clinical staff, such as physicians, nurses, pharmacists, and dentists. Vigorous and extensive study designs have not been developed to assess the competence of health workers in fulfilling the needs of a specific population [12]. This, however, is imperative for the governance of an efficient healthcare system. With regard to menstrual management, the role played by healthcare workers has been studied less commonly and has always been cited as unsupportive or as a rare source of information [3,4]. Considering this, the secondary objective of our study was to compare the aforementioned parameters in healthcare workers and to determine the need or the lack thereof of their counselling in this context.

## Materials and methods

A descriptive cross-sectional study was conducted between October 2019 and January 2020 to compare the beliefs and practices regarding menstruation between healthcare workers and the general female population in Karachi, Pakistan. It was a questionnaire-based study with a sample size of 353 participants. The data was collected from two tertiary care hospitals, i.e. Dr. Ruth K. M. Pfau Civil Hospital Karachi and Dow University Hospital Ojha.

The sample size was calculated using OpenEpi.com sample size calculator, version 3.01 [13]. Using an anticipated frequency (p) of 67.1%, margin error of 5%, and a confidence interval (CI) of 95%, minimal size of the sample was computed to 340 [13,14]. The sample was divided into two groups, health workers and women visiting the gynecologic and obstetrics out-patient department (OPD) of the two hospitals. 400 women were approached to participate in the study, 200 from the general population and 200 healthcare workers. Among those women, 353 consented to participate in the study, and the response rate was calculated to be 88.25%.

Females having undergone menarche and those of reproductive age were included in the study, while those women falling in the category of precocious puberty and undergone menopause, having any chromosomal aberrations, undergone hysterectomy, facing severe language barrier, or any declared mental and psychological disorder were excluded. Resident trainees, physicians, nurses, and house officers (interns) were subsumed under healthcare workers.

Data was collected through a self-reported, self-administered questionnaire with a total number of 38 questions. There was one open-ended and 37 close-ended questions. In order to ensure the reliability and validity of the questionnaire and to prevent ambiguities, a pilot study was conducted where 15 health workers and populace meeting the inclusion criteria were asked to fill the questionnaire and their feedback was incorporated to upgrade the final version. Expert opinion of a senior gynecologist was also gained to refine and finalize the questionnaire. The participation of candidates was voluntary, and permission was taken through a written informed consent form at the beginning of the questionnaire. Personal information was kept confidential and anonymous. Questions were translated in the national Pakistani language and lingua franca, Urdu, to help participants who were unable to read or understand English language, and assistance was given by investigators if the respondents faced difficulties.

The questionnaire was subdivided into four parts: sociodemographic characteristics, knowledge of the participants regarding menstruation, reactions and practices pertaining to menstruation, and problems and challenges faced by women during menstruation. The socio-demographic characteristics of the participants included age, marital status, educational status, mother’s educational status, family income, and religion. The second section regarding knowledge of the participants included questions such as source of knowledge related to menstruation, awareness regarding tampons and menstrual cups, knowledge about menstruation at the time of menarche, and awareness regarding the cause of infrequent menses.

Third section of the questionnaire included questions associated with general menstrual hygiene practices like taking bath, reasons for avoiding bath, materials used while taking bath, exercise, washing groin area, and restriction of activities. It also interviewed women regarding sanitary choices to avoid staining (for example cloth, pad, tampon, and cups), the reasons behind them, ways of disposing of them, and the number of times the absorbent was changed. Moreover, this section covered questions pertaining to reactions of women when they menstruated for the first time (scared, guilty or confused), and if they were openly able to discuss any gynecological issues they were facing. 

The last section of the questionnaire listed the problems and challenges faced by participants during menstruation such as going out of their homes, avoiding certain food items, refraining from taking medicines, unavailability of sanitary pads in their institute/workplace, and experiencing any other issues like bloating, anxiety, nausea, etc., or having complaints of a bad odor during periods. The participants were also asked if they had ever faced a few of the common gynecological complaints and if they sought any treatment for it. 

Data was tabulated in Statistical Package for the Social Sciences (SPSS version 24.0, IBM Corp., Armonk, NY, US) and presented in the form of mean, frequencies, and percentages. In accordance with the objectives of the study, descriptive analysis was performed. Chi-square test was applied for categorical variables, while independent sample t test was applied to analyze continuous variables. P-values less than 0.05 were considered to be statistically significant.

## Results

The current study was conducted employing 353 females, amongst which 176 were from the general population and 177 were healthcare workers. As shown in Table 1 below, the mean age of the general population was 29.89 ± 9.17 with the age range being 14-50 years, while the mean age of the healthcare workers was 30.54 ± 6.52 with the age range being 23-54 years. Most of the females amongst the general population were uneducated (26.1%), while the majority of the healthcare workers were graduates (62.1%). Similarly, 51.1% of the general population's females’ mothers were uneducated while 58.8% of healthcare workers’ mothers were graduates. The majority of respondents from both categories were Muslims and lived in a brick-built house. 62.5% of the general population was observed to have family income of below 40,000 Pakistani Rupee (PKR) and 55.9% of healthcare workers had a family income between 40,000-100,000 PKR. A large fraction of females from the general population were married (65.9%) while the majority of healthcare workers were single (66.7%).

**Table 1:** Socio-demographic characteristics of the participants SD: standard deviation; PKR: Pakistani Rupee ^a^ Calculated using Chi-square test; P-value of <0.05 considered statistically significant ^b^ Number of females from general population: 176; number of healthcare workers: 177

Sociodemographic variables	General population, N (%)	Healthcare workers, N (%)	P-value
Mean age (years) ± SD	29.89 ± 9.17	30.54 ± 6.52	0.447
Age range (years)	14-50	23-54	
Education			0.000
Postgraduate	4 (2.3)	67 (37.9)	
Graduate	42 (23.9)	110 (62.1)	
Intermediate	29 (16.5)	0 (0)	
Secondary	34 (19.3)	0 (0)	
Primary	21 (11.9)	0 (0)	
Uneducated	46 (26.1)	0 (0)	
Education of mother			0.000
Postgraduate	6 (3.4)	17 (9.6)	
Graduate	20 (11.4)	104 (58.8)	
Intermediate	16 (9.1)	30 (16.9)	
Secondary	20 (11.4)	18 (10.2)	
Primary	24 (13.6)	6 (3.4)	
Uneducated	90 (51.1)	2 (1.1)	
Total family income per month			0.000
Above PKR 100,000	18 (10.2)	56 (31.6)	
Between PKR 40,000-100,000	48 (27.3)	99 (55.9)	
Below PKR 40,000	110 (62.5)	22 (12.4)	
House Structure			0.000
Pakka (brick-built)	104 (59.1)	172 (97.2)	
Semi Pakka (tin roofing or sheets)	35 (19.9)	5 (2.8)	
Kaccha (no bricks)	37 (21.0)	0 (0)	
Marital Status			0.000
Married	116 (65.9)	57 (32.2)	
Widowed	2 (1.1)	2 (1.1)	
Divorced	1 (0.6)	0 (0)	
Single	57 (32.4)	118 (66.7)	
Religion			0.645
Islam	172 (97.7)	170 (96.0)	
Christianity	2 (1.1)	4 (2.3)	
Hinduism	2 (1.1)	3 (1.7)	

Knowledge of the participants regarding menstruation and their awareness at the time of menarche was assessed as shown in Table 2 below. It was found that amongst both categories, the most common source of information regarding menstruation were the mothers of participants (58.5% for the general population and 53.1% for healthcare workers). Only about one-third of the participants from both groups (28.4% from the general population and 29.4% from healthcare workers) were completely aware of menstruation and knew the proper course of action at the time of menarche. Data shows 88.6% of females from the general population and 57.6% of the healthcare workers were unaware of menstrual cups. While 53.1% of healthcare workers were observed to be aware of tampons, 84.1% of females from the general population were found to be lacking in their knowledge of such products. Infrequent menstruation was assumed to be a sign of pregnancy in 58% of the general population, while only 23.2% of healthcare workers believed such assumptions to be true.

**Table 2:** Knowledge of the participants regarding menstruation ^a^ Calculated using Chi-square test; P-value of <0.05 considered statistically significant ^b^ Number of females from general population: 176; number of healthcare workers: 177

	General Population, N (%)	Healthcare workers, N (%)	P-value
Source of general overview and do’s and don'ts of menstruation			0.000
Mother	103 (58.5)	94 (53.1)	
Grandmother	9 (5.1)	6 (3.4)	
Teachers	6 (3.4)	12 (6.8)	
Relatives or siblings	28 (15.9)	10 (5.6)	
Friends	14 (8.0)	30 (17.0)	
Self-knowledge (TV, Books, Internet)	7 (4.0)	22 (12.4)	
No one	9 (5.1)	3 (1.7)	
Knowledge at menarche			0.000
I was already aware of periods, knew how to prevent staining my clothes and knew how to properly place the cloth/ pad	50 (28.4)	52 (29.4)	
I was already aware of periods, knew how to prevent staining my clothes but had no idea of how to properly place the cloth/pad	40 (22.7)	48 (27.1)	
I was aware of periods but didn’t know how to prevent staining my clothes	24 (13.6)	39 (22.0)	
I thought I had some disease/infection	34 (19.3)	8 (4.5)	
I thought I had suffered an injury	14 (8.0)	7 (4.0)	
I thought I was pregnant	1 (0.6)	1 (0.6)	
I was not aware at all and sought elders	13 (7.4)	22 (12.4)	
Are you aware of what a tampon is?			0.000
Yes	28 (15.9)	94 (53.1)	
No	148 (84.1)	83 (46.9)	
Are you aware of what a menstrual cup is?			0.000
Yes	20 (11.4)	75 (42.4)	
No	156 (88.6)	102 (57.6)	
Do you believe that infrequent menses is a sign of pregnancy?			0.000
Yes	102 (58.0)	41 (23.2)	
No	74 (42.0)	136 (76.8)	

**Figure 1:**
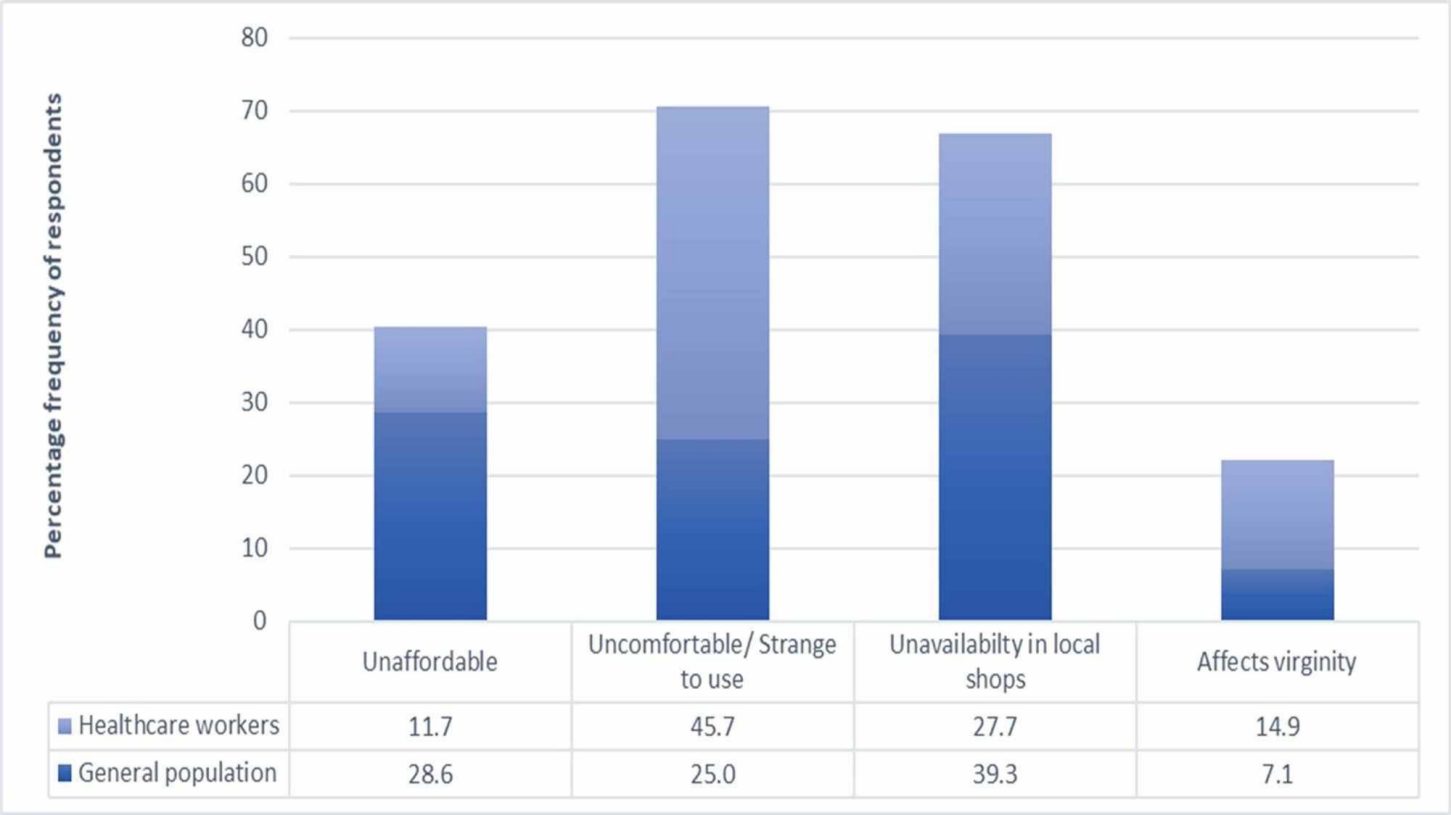
Reasons for not using tampons amongst general population and healthcare workers

The responses of females from both subgroups who were aware of tampons and were questioned regarding the reasons for not using them are illustrated in Figure 1. The most common reason amongst healthcare workers was found to be that it is “uncomfortable/ strange to use”, while the majority of the general population responded that it was “unavailable in local shops”. Lack of affordability due to the high cost of the product was found to be significantly associated with the general population as compared to healthcare workers (P-value = 0.049).

The respondents were further questioned regarding their reactions and practices pertaining to menstruation as shown in Table 3. Majority of the respondents amongst the general population (65.9%) and healthcare workers (75.1%) were able to talk freely about any gynecological issues they were facing. An astounding finding in our study was that most women from both categories (77.8% of general populace and 66.1% of healthcare workers) avoided bathing during menstruation. The females were further questioned about the number of days they avoided bathing, where 48.7% of healthcare workers responded that they avoid it for the first day and 44.5% of general population avoided it for the first three days of menstruation. Data showed that 68.8% of general population and 88.7% of healthcare workers responded that they wash the groin after urination or defecation. Water and soap were the most common means of taking a bath when menses ended.

**Table 3:** Reactions and practices of the participants pertaining to menstruation ^a^Calculated using Chi-square test; P-value of <0.05 considered statistically significant ^b^Number of females from general population: 176; number of healthcare workers: 177

	General population, N (%)	Healthcare workers, N (%)	P-value
Are you able to talk freely with your mother or any relatives about any gynecological issues?			0.057
Yes	116 (65.9)	133 (75.1)	
No	60 (34.1)	44 (24.9)	
Do you avoid bathing during menses?			0.014
Yes	137 (77.8)	117 (66.1)	
No	39 (22.2)	60 (33.9)	
If yes, how many days do you avoid bathing during menses?			0.013
During the first day	44 (32.1)	57 (48.7)	
During the first three days	61 (44.5)	45 (38.5)	
During all days	32 (23.4)	15 (12.8)	
Do you avoid washing the groin after urination/defecation?			0.000
Yes	55 (31.2)	20 (11.3)	
No	121 (68.8)	157 (88.7)	
Do you stop doing exercise during menses?			0.175
Yes	24 (13.6)	27 (15.3)	
No	40 (22.7)	54 (30.5)	
I don’t do exercise regularly	112 (63.6)	96 (54.2)	
Do you have any restrictions of activity during menses?			0.002
Yes	94 (53.4)	66 (37.3)	
No	82 (46.6)	111 (62.7)	
If yes, which ones?			0.015
Climbing stairs	31 (33.0)	12 (18.2)	
Lifting weight	41 (43.6)	44 (66.7)	
Walking	22 (23.4)	10 (15.1)	
What were your feelings when you menstruated for the first time?			
Scared			0.043
Yes	74 (42.0)	56 (31.6)	
No	102 (58.0)	121 (68.4)	
Guilty			0.493
Yes	16 (9.1)	20 (11.3)	
No	160 (90.9)	157 (88.7)	
Upset			0.045
Yes	32 (18.2)	48 (27.1)	
No	144 (81.8)	129 (72.9)	
Anxious			0.109
Yes	53 (30.1)	40 (22.6)	
No	123 (69.9)	137 (77.4)	
Normal			0.003
Yes	20 (11.4)	41 (23.2)	
No	156 (88.6)	136 (76.8)	
Confused			0.408
Yes	66 (37.5)	74 (41.8)	
No	110 (62.5)	103 (58.2)	
Miserable			0.005
Yes	12 (6.8)	29 (16.4)	
No	164 (93.2)	148 (83.6)	
Shame			0.000
Yes	43 (24.4)	12 (6.8)	
No	133 (75.6)	165 (93.2)	
Excited/Delighted			0.191
Yes	8 (4.5)	14 (7.9)	
No	168 (95.5)	163 (92.1)	
Which of these do you use during menstruation to avoid staining your clothes?			0.000
Cloth	53 (30.1)	15 (8.5)	
Pads	93 (52.8)	151 (85.3)	
Cloth and pads	17 (9.7)	9 (5.1)	
Tampon	3 (1.7)	2 (1.1)	
Tissue paper	1 (0.6)	0 (0)	
Cotton	8 (4.5)	0 (0)	
Menstrual cup	1 (0.6)	0 (0)	
For those using cloth, is the cloth:			0.984
Torn from a new fabric	29 (41.4)	10 (41.7)	
Torn from an old worn out shirt	41 (58.6)	14 (58.3)	
How many times do you change the menstrual product?			0.074
4 or more times per day	28 (16.0)	17 (9.6)	
2-3 times per day	115 (65.3)	127 (71.8)	
Once per day	24 (13.6)	30 (16.9)	
Use 1 for almost 2 days	9 (5.1)	3 (1.7)	
How do you dispose of the menstrual products after using them?			0.000
Bury it	12 (6.8)	1 (0.6)	
Burn it	10 (5.7)	1 (0.6)	
Flush it in the toilet	2 (1.1)	1 (0.6)	
Throw it in waste	133 (75.6)	170 (96.0)	
Throw it in sea	4 (2.3)	2 (1.1)	
Store it for further use	15 (8.5)	2 (1.1)	
What do you use while taking a bath after your period ended?			0.010
Water only	16 (9.1)	8 (4.5)	
Water and soap	136 (77.3)	158 (89.3)	
Water and antiseptic	24 (13.6)	11 (6.2)	

Data revealed that 53.4% of general populace was found to have certain restrictions of activity during menstruation, amongst which the most common was lifting weight. The feelings of the women at the time of menarche were also questioned. About 42% of women from the general population responded that they were scared while 41.8% of healthcare workers were confused at the time of menarche. The most common product used by women from both categories (52.8% of general population and 85.3% of healthcare workers) at the time of menstruation was sanitary pads, followed by cloth (30.1% of general population and 8.5% of healthcare workers). Amongst those using cloth, 58.6% of the general population and 58.3% of healthcare workers were found to be using the fabric torn from an old, worn-out shirt. Majority of women from both categories (65.3% of the general populace and 71.8% of healthcare workers) changed their menstrual products two to three times per day. A large fraction of females from both categories disposed of the menstrual products by throwing them in the waste.

**Figure 2:**
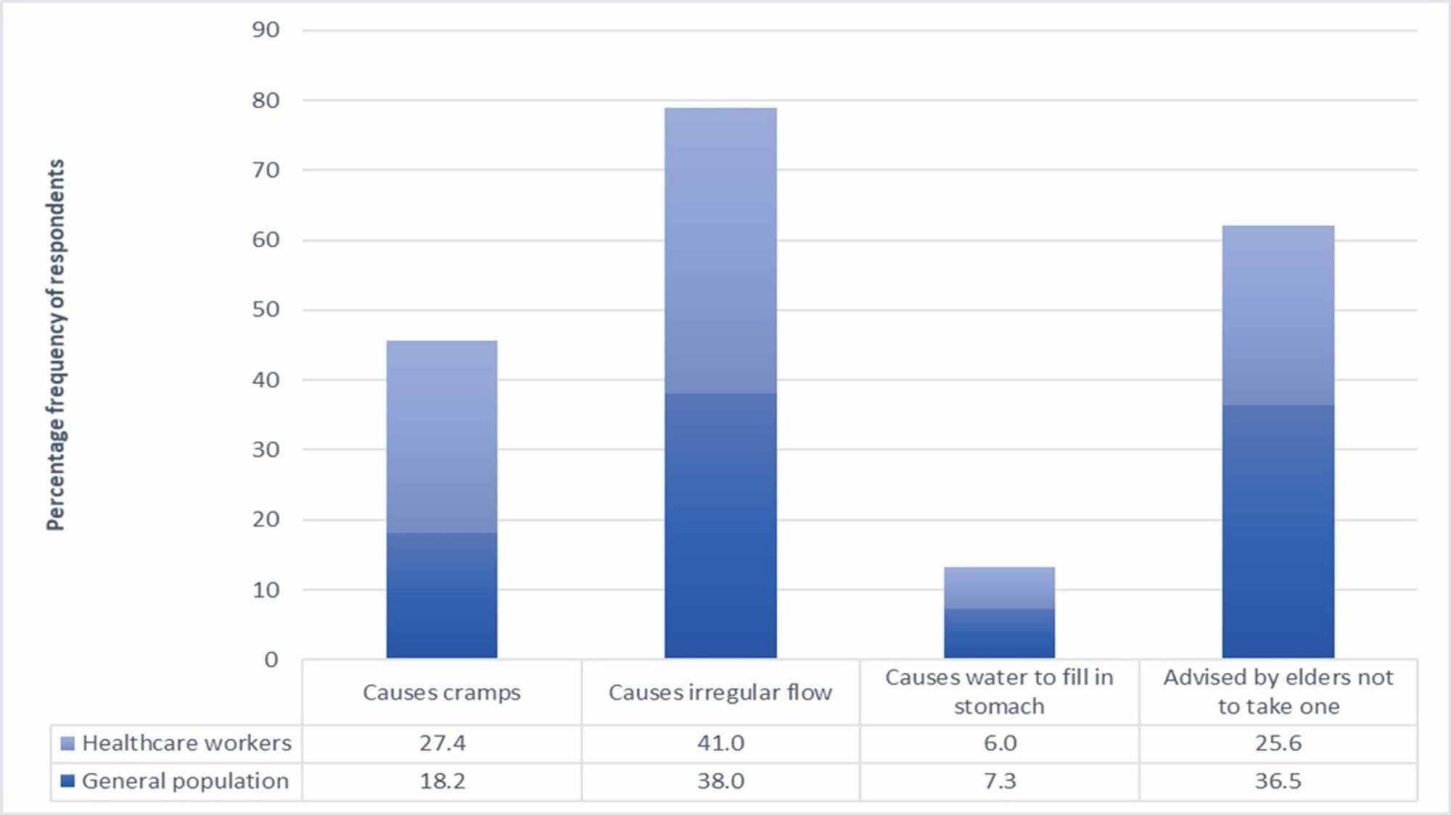
Reasons for avoidance of bathing amongst general population and healthcare workers

The women were interviewed regarding the reasons they take bath or avoid it during menses. As shown in Figure 2 below, the most common reason for avoidance of bathing was found to be that "it can cause irregular flow", amongst both the general population and healthcare workers. None of these reasons were found to be statistically significant between general population and healthcare workers (P-value > 0.05).

**Figure 3:**
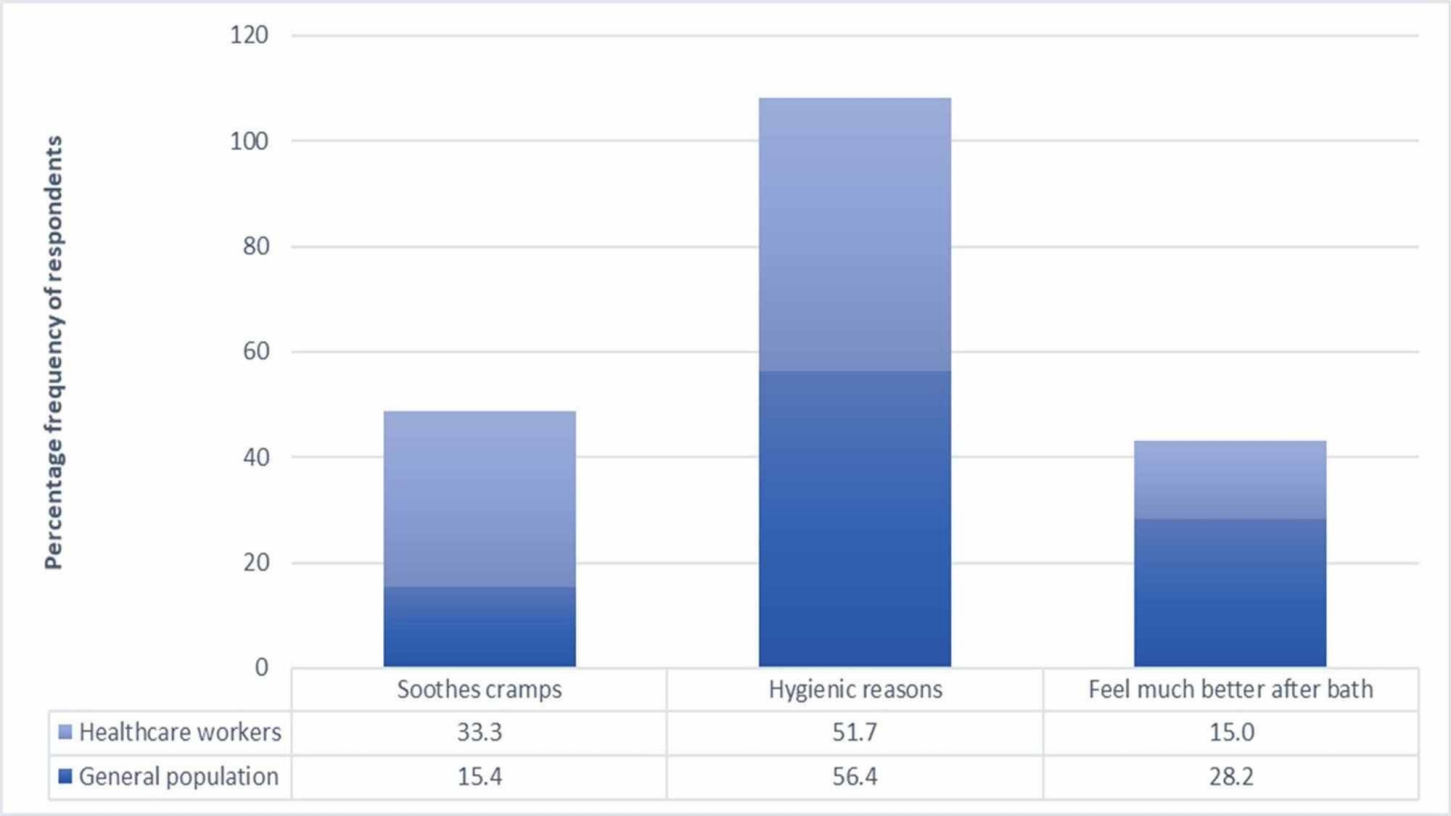
Reasons for taking bath amongst general population and healthcare workers

As shown in Figure 3 below, the most common reason for taking bath was found to be “hygienic reasons” amongst both the general population and healthcare workers. The practice of taking bath to soothe cramps was found to be significantly higher in healthcare workers than in the general population (P-value = 0.047).

The respondents of the study were questioned regarding the problems and challenges faced by them during menstruation as shown in Table 4. Around 64.2% of the women from the general population abstained from eating certain food items whereas only 28.8% of healthcare workers were found to be following this practice. The women were questioned about whether they experience any premenstrual symptoms, amongst which most women from the general population experienced fatigue (47.7%), while majority of women from healthcare workers experienced mood swings/irritability (48.6%). Around 32.4% of women from the general population and 26% of healthcare workers avoided taking any medicine during menstruation due to the innate fear that their cycle would be disturbed. Majority of women from both factions (54% of the general populace and 65% of healthcare workers) went out of their homes without any inhibitions. However, a comparatively higher number of women from the general population (5.7%) avoided going out of their homes because they feared evil spirits/black magic.

**Table 4:** Problems and challenges faced by participants during menstruation ^a^ Calculated using Chi-square test; P-value of <0.05 considered statistically significant ^b^ Number of females from general population: 176; number of healthcare workers: 177

	General population, N (%)	Healthcare workers, N (%)	P-value
Do you abstain from eating certain food items during menses?			0.000
Yes	113 (64.2)	51 (28.8)	
No	63 (35.8)	126 (71.2)	
Which one of these complains do you have during menses?			
Tension or anxiety			0.711
Yes	69 (39.2)	66 (37.3)	
No	107 (60.8)	111 (62.7)	
Poor concentration			0.200
Yes	32 (18.2)	42 (23.7)	
No	144 (81.8)	135 (76.3)	
Abdominal bloating			0.000
Yes	31 (17.6)	68 (38.4)	
No	145 (82.4)	109 (61.6)	
Change in sexual desires			0.160
Yes	37 (21)	27 (15.3)	
No	139 (79.0)	150 (84.7)	
Breast tenderness			0.001
Yes	17 (9.7)	39 (22.0)	
No	159 (90.3)	138 (78.0)	
Crying spells			0.041
Yes	20 (11.4)	34 (19.2)	
No	156 (88.6)	143 (80.8)	
Hair fall or dandruff			0.195
Yes	20 (11.4)	13 (7.3)	
No	156 (88.6)	164 (92.7)	
Acne			0.498
Yes	40 (22.7)	35 (19.8)	
No	136 (77.3)	142 (80.2)	
Mood swings or irritability			0.260
Yes	75 (42.6)	86 (48.6)	
No	101 (57.4)	91 (51.4)	
Joint or muscle pain			0.000
Yes	65 (36.9)	35 (19.8)	
No	111 (63.1)	142 (80.2)	
Constipation or diarrhea			0.032
Yes	21 (11.9)	36 (20.3)	
No	155 (88.1)	141 (79.7)	
Appetite changes or food cravings			0.726
Yes	39 (22.2)	42 (23.7)	
No	137 (77.8)	135 (76.3)	
Headache			0.008
Yes	52 (29.5)	31 (17.5)	
No	124 (70.5)	146 (82.5)	
Nausea or vomiting			0.246
Yes	25 (14.2)	18 (10.2)	
No	151 (85.8)	159 (89.8)	
Trouble falling asleep			0.089
Yes	37 (21.0)	25 (14.1)	
No	139 (79.0)	152 (85.9)	
Fatigue			0.001
Yes	84 (47.7)	53 (29.9)	
No	92 (52.3)	124 (70.1)	
Abdominal pain			0.300
Yes	68 (38.6)	78 (44.1)	
No	108 (61.4)	99 (55.9)	
Social withdrawal			0.025
Yes	46 (26.1)	29 (16.4)	
No	130 (73.9)	148 (83.6)	
Do you avoid taking any medicine during menses? (fear that the menses will stop)			0.186
Yes	57 (32.4)	46 (26.0)	
No	119 (67.6)	131 (74.0)	
Regarding going out of your home during periods:			0.071
I avoid going out because I experience a lot of pain	35 (19.9)	33 (18.6)	
I avoid going out because I feel my energy gets low during periods	15 (8.5)	12 (6.8)	
I avoid going out because I’m scared of the evil spirits/black magic	10 (5.7)	2 (1.1)	
I avoid going out because I’m scared of staining my clothes accidentally	21 (11.9)	15 (8.5)	
I go out of my home without any inhibitions	95 (54.0)	115 (65.0)	
Do you feel that you have a bad odor during periods?			0.000
Yes	109 (61.9)	57 (32.2)	
No	67 (38.1)	120 (67.8)	
What is the reason for using cloth/tissue paper/cotton?			0.920
Pads are uncomfortable to use	18 (29.0)	6 (40.0)	
Pads are unavailable in nearby shops	11 (17.7)	3 (20.0)	
Pads are unaffordable	22 (35.5)	4 (26.6)	
I feel shy to buy pads because the shopkeepers are male	6 (9.7)	1 (6.7)	
I feel shy asking my family men to buy pads for me	5 (8.1)	1 (6.7)	
Have you ever had?			
Any reproductive tract infection			0.000
Yes	41 (23.3)	12 (6.8)	
No	135 (76.7)	165 (93.2)	
Foul-smelling/abnormal discharge			0.379
Yes	23 (13.1)	29 (16.4)	
No	153 (86.9)	148 (83.6)	
Painful sores on pubic area			0.745
Yes	18 (10.2)	20 (11.3)	
No	158 (89.8)	157 (88.7)	
Rashes on pubic area			0.001
Yes	65 (36.9)	38 (21.5)	
No	111 (63.1)	139 (78.5)	
Pain or cramps during periods			0.044
Yes	48 (27.3)	66 (37.3)	
No	128 (72.7)	111 (62.7)	
Early period			0.006
Yes	22 (12.5)	42 (23.7)	
No	154 (87.5)	135 (76.3)	
Delayed period			0.001
Yes	28 (15.9)	54 (30.5)	
No	148 (84.1)	123 (69.5)	
Missed/infrequent period			0.568
Yes	35 (19.9)	31 (17.5)	
No	141 (80.1)	146 (82.5)	
Light flow			0.106
Yes	34 (19.3)	47 (26.6)	
No	142 (80.7)	130 (73.4)	
Heavy flow			0.624
Yes	36 (20.5)	40 (22.6)	
No	140 (79.5)	137 (77.4)	
No issues			0.231
Yes	15 (8.5)	22 (12.4)	
No	161 (91.5)	155 (87.6)	
Treatment sought for gynecological issues?			0.408
Yes	51 (29.0)	57 (32.2)	
No	110 (62.5)	98 (55.4)	
No issues	15 (8.5)	22 (12.4)	
Are there sanitary pads available in your institute/college/workplace?			0.000
Yes	89 (50.6)	109 (61.6)	
No	56 (31.8)	68 (38.4)	
I stay at home	31 (17.6)	0 (0)	

A significantly higher number of women from the general population (61.9%) felt that they have a bad odor during menses while most women from healthcare workers (67.8%) refused any such thing. The women using cloth, tissue paper or cotton were questioned about the reasons for not using pads amongst which the most common reason in the general population was the lack of affordability of the product (35.5%) while the majority of the healthcare workers (40%) found the product to be uncomfortable.

The women were further interviewed regarding experiencing any problem related to their reproductive health. Significantly higher number of women from the general population had experienced reproductive tract infections (23.3%) and rashes on pubic area (36.9%) as compared to healthcare workers. Similarly, a significantly higher number of healthcare workers had experienced delayed or early periods (30.5% and 23.7% respectively) and pain or cramps during periods (37.3%) as compared to the general population. Despite facing these issues, only a small fraction of women from both groups sought proper treatment (29% from the general populace and 32.2% of healthcare workers). Women were also interviewed regarding the availability of sanitary pads in their institute/college/workplace to which a significantly higher number of healthcare workers (61.6%) replied in affirmative as compared to the general population (50.6%).

**Figure 4:**
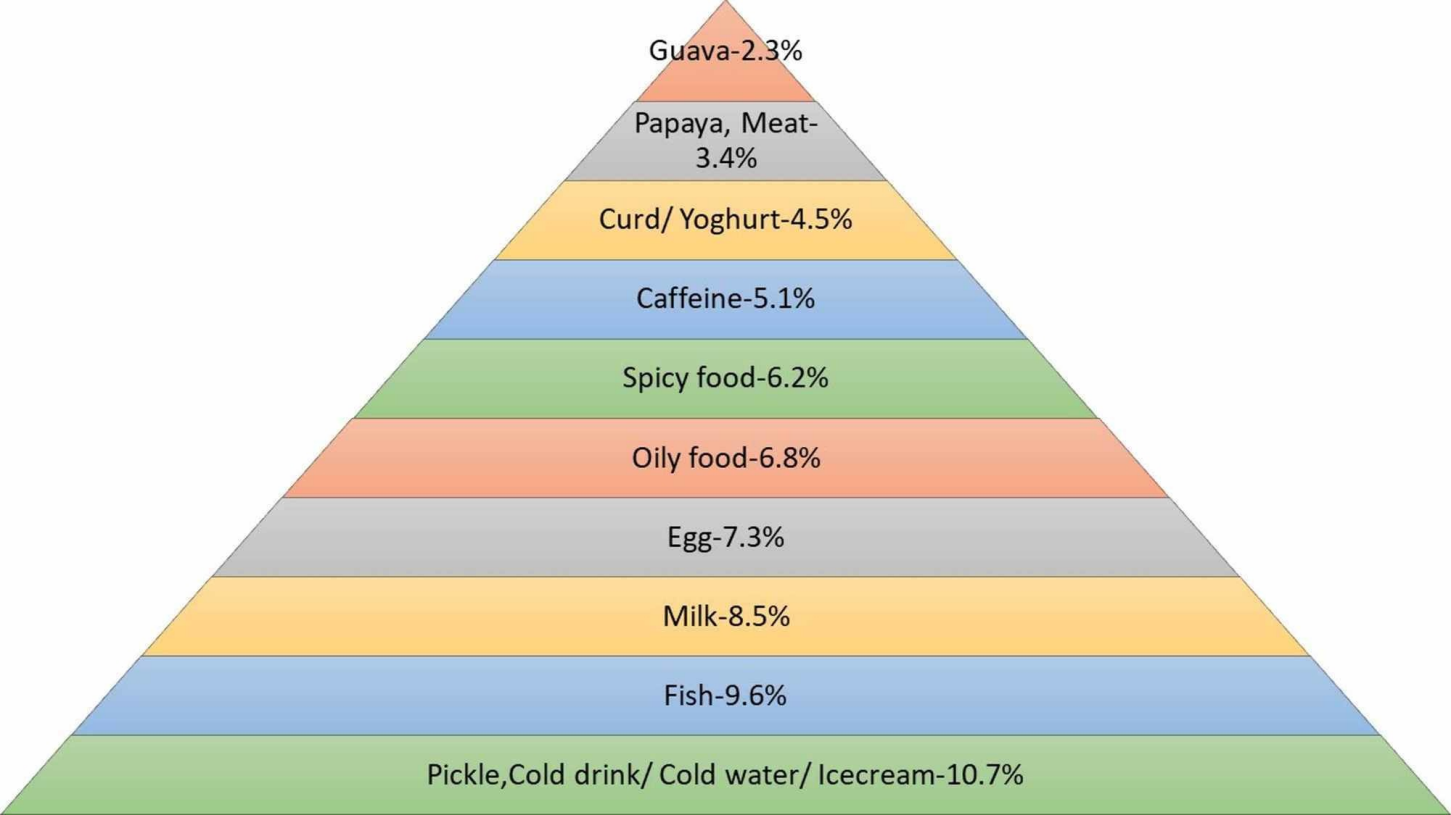
Food items avoided by healthcare workers during menstruation

The women of both groups were questioned regarding the food items they abstained from during menstruation. As shown in Figure 4 below, pickle, cold drink, cold water, and ice cream were found to be the most common food items avoided by healthcare workers during menstruation.

**Figure 5:**
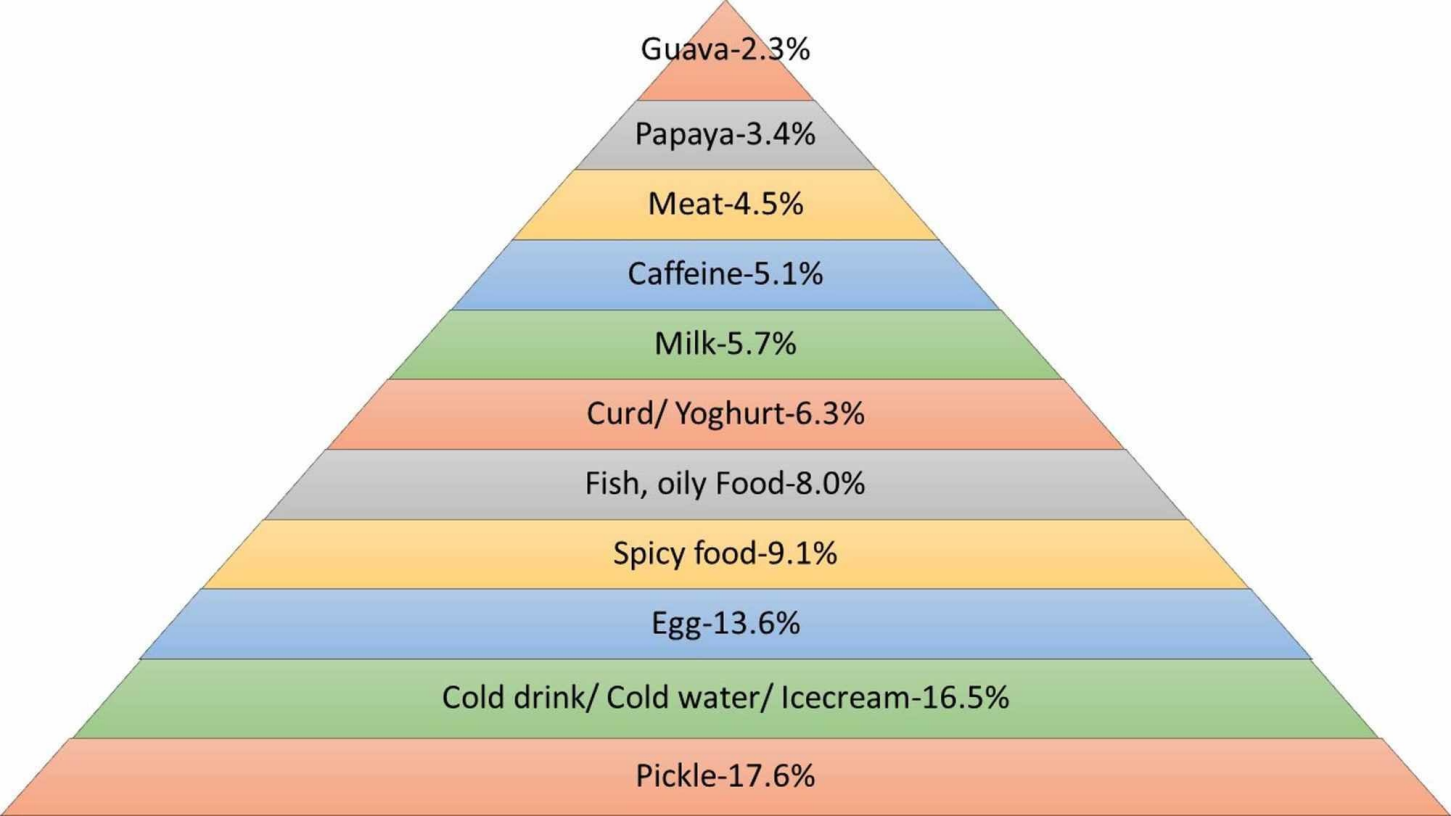
Food items avoided by general population during menstruation

Majority of women from the general population responded that pickle was the most common food item avoided during menstruation as illustrated in Figure 5 below. Cold drink, cold water, and ice cream were found to be the second most common food items to be refrained from, while guava was found to be the least common food item to be avoided.

**Figure 6:**
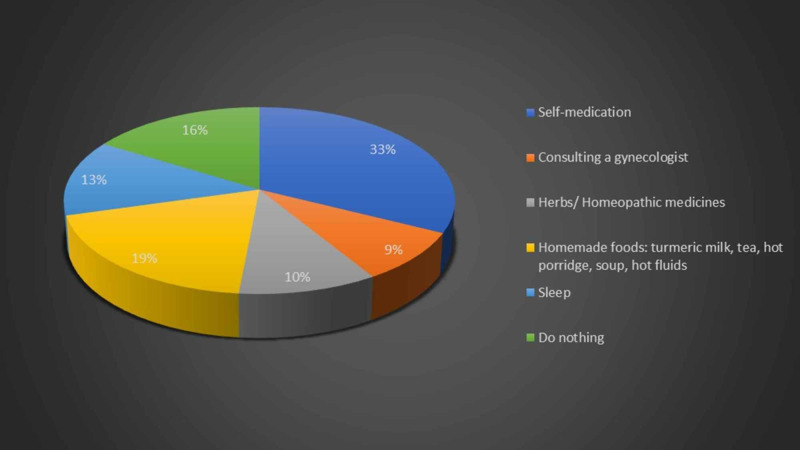
Relieving factors for abdominal pain employed by general population

Around 19.9% of women from the general population and 18.6% of healthcare workers avoided going out of their homes during periods because they experienced a lot of pain. Moreover, 38.6% women from the general population and 44.1% of healthcare workers experienced abdominal pain during menses. Hence, the women were questioned regarding the strategies they employed to relieve abdominal pain during menses. As shown in Figure 6 below, self-medication was found to be the most common relieving factor amongst the general population followed by homemade foods such as turmeric milk, tea, hot porridge, soup, and hot fluids.

**Figure 7:**
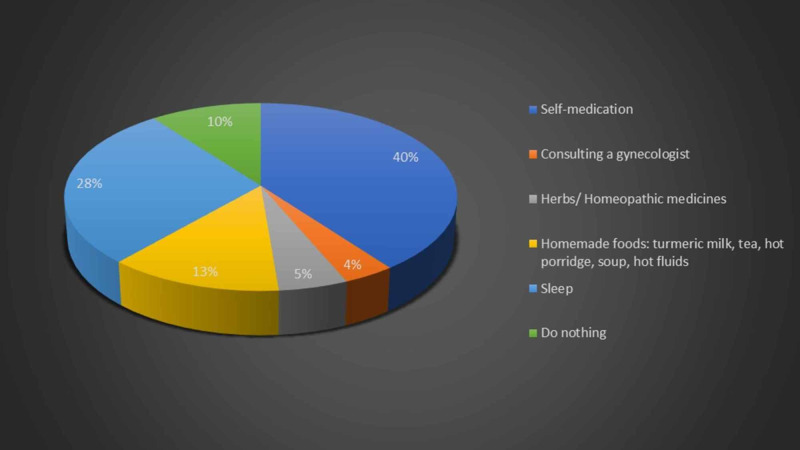
Relieving factors for abdominal pain employed by healthcare workers

A large faction of women from healthcare workers opted self-medication as a remedy for pain during menstruation as illustrated in Figure 7. A significantly higher number of healthcare workers counteracted the pain by sleeping as compared to the general population (P-value = 0.027).

## Discussion

Menstrual hygiene implies understanding the basic facts underlying the menstrual cycle and to manage it with dignity and comfort. Access to sanitary products, adequate sanitation, and dissemination of information amidst the stigma, is a challenge for a developing country like Pakistan. In this study, we have assessed the knowledge, attitude, practices, and challenges regarding menstruation faced by the women in Karachi. We have also compared these parameters in healthcare workers to assess how their contribution can bridge the gap.

In concordance to the studies conducted in Quetta, India, and the USA, mothers were the most frequently cited source of information by both the healthcare workers and general population in our study [1,8,15]. In our study, the educational status of mothers, of healthcare workers, was considerably better than that of the general population (P-value = 0.00). Upashe et al. identified a positive correlation between the educational status of the mother and good menstrual hygiene practice [16]. Mothers with poor knowledge of the physiology of menses will endorse and propagate unhygienic practices. Unless we bridge the knowledge gap prevalent among mothers and destigmatize menstruation, we cannot expect proper menstrual hygiene management by young girls at the time of menarche. Although menarche should be celebrated as a sign of womanhood and fertility, it is marred by the taboo associated with it in our socio-cultural set-up. Negative connotations are tied to menstruation, labelling it as dirty or impure blood [8]. For a topic that is draped in secrecy, acquiring information and seeking support becomes difficult. Thus, the majority of the participants in our study recalled that they were most likely scared and confused at the time of menarche. In alignment with our study, being unprepared owing to similar reasons, young girls from impoverished areas across the USA had negative experiences at menarche [15]. In our study, a significantly higher number of participants from the general population presumed infrequent menstruation to be a sign of pregnancy (P-value = 0.00). Infrequent menstruation, apart from being associated with pregnancy, is also a sign that needs to be investigated and clinically dealt with as it may be due to conditions like polycystic ovarian syndrome (PCOS) and low body mass index [17]. Hence, healthcare workers should enlighten young girls among the general population about menstrual irregularities and their impact on their gynecological health. 

As healthcare workers had a better family income than the general population, the majority of them could afford to use pads. To avoid friction in their household, some participants in our study used cloth torn from a worn-out fabric. Women who use cloth are twice as likely to acquire bacterial vaginosis as compared to women who use nothing [18]. In a study conducted in Delhi, India it was observed that women reusing the same cloth are more likely to get infected with bacterial vaginosis [19]. The cost of sanitary pads was the main deterrent as reported by other studies conducted in India, Tanzania, and Uganda [6,20,21]. In a study conducted in Ethiopia, girls who earned pocket money from their parents could afford sanitary napkins and were three times more likely to have good menstrual practice [16]. A study revealed that some women in Kenya had to resort to transactional sex to afford menstrual products [22]. Surprisingly, the conditions in the developed world are far from satisfactory. A survey conducted in Canada revealed that one-third of menstruators had difficulties managing their budgets to be able to afford menstrual products [23]. In another study, women surveyed in Karachi, Pakistan gave a similar reason as some of our study participants for not using sanitary pads, i.e., they were uncomfortable and caused rashes [24]. Some of our study participants (9.7% of the general population and 6.7% of healthcare workers) hesitated to buy menstrual products from male shopkeepers, hence, again indicating the dire need to address the taboo. Even male counterparts can play a role in guaranteeing a safe menstrual experience to women. It is highly recommended that, at a household level, men should prioritize menstrual needs and personal hygiene of the women by financing the menstrual products. Sanitary pads and tampons are deemed as a ‘luxury’ and not a basic necessity and thus women have no other choice than to use and reuse the same cloth. Due to this, women hailing from low socio-economic backgrounds, have to compromise on hygiene and are particularly vulnerable to the repercussions of poor practices [25].

As healthcare workers had better awareness of tampons as compared to the general population, they can play a role in disseminating information about potential alternatives to menstrual pads. Tampons are widely used in the Western world as they allow unrestricted continuance of sports and work during menstruation. In our study, the cost of tampons proved to be a greater deterrent for the general population than healthcare workers (P-value = 0.049). It was surprising to find that 57.6% of healthcare workers were unaware of menstrual cups. A review article suggested that menstrual cups can be a very safe option even for low-income countries as they salvage the cost of purchasing pads or soap and can be used as a suitable alternative in the setting of inadequate sanitation facilities [26]. Menstrual cups are a long-term investment since they can be reused multiple times if sanitized properly, and eventually, save the cost of purchasing pads. Being able to collect more blood than tampons, they are preferred by women with menorrhagia [25]. The only deterrent is the lack of community and peer support. Healthcare workers should be well acquainted with these products so that they can encourage their local use.

It was surprising to find that healthcare workers and the general population avoided bathing on the first and the first three days of menstruation respectively, owing to the similar belief that it caused irregular menstrual flow, followed by the instructions from elders. These findings are in alignment with studies conducted in Quetta and Karachi where similar reasons were reported for not bathing [8,24]. An Egyptian study revealed some other harbored superstitions associated with bathing such as backache, hair fall, skin maceration, heavy menstrual bleeding, and discontinuation of menstrual flow [24]. Although there is no medical or scientific basis of these myths, even healthcare workers harbor these beliefs, similar to that of the general population (P-value > 0.05). Healthcare workers should reevaluate these practices as they are more likely to endorse and recommend similar practices to the general masses. In fact, not taking a bath translates as a compromise in hygiene that can culminate in reproductive tract infections [27]. Contrary to a study conducted in Quetta, where an overwhelming majority of the study participants did not take a bath but still used soap and water to clean their genitals, 31.2% of the general populace and 11.3% of healthcare workers in our study avoided washing their groin area after urination or defecation [8].

The majority of our study participants changed their pads/cloth two to three times per day and disposed of it by throwing it in the waste. Hygiene guidelines recommend changing absorbents every two to six hours dependent on blood flow [7]. A significantly higher number of women from the general population felt that they have a bad odor during menses (P-value= 0.00) and had experienced reproductive tract infection (P-value= 0.00) and rashes on the pubic area (P-value= 0.001) as compared to healthcare workers. This can be attributed to poor knowledge that translates into poor practices. Infrequently changing the cloth or pad that is making use of just two or three pads during a whole period lasting three or more days can result in bad odor. A review article highlighted the finding that bad odor contributed to fear, embarrassment, and distress among school-going girls [4]. Limited availability of soap and water deters some women from low socio-economic backgrounds, to ensure frequent washing of groin during menses [4]. Despite facing these issues, only a small fraction of women from both groups sought proper treatment (29% of the general populace and 32.2% of healthcare workers), which can eventually lead to exacerbation of the pubic sores and infection. It has been suggested that women with dysmenorrhea or other menstrual disorders were often hesitant to discuss matters pertaining to sexual health owing to their warped cultural values and many found the healthcare providers to be unsupportive [4].

More than half of our respondents from the general population abstained from eating certain food items especially pickles and cold beverages, followed by eggs. Only 28.8% of healthcare workers abstained from eating similar food items. Our findings are in alignment with another study conducted in Karachi but in stark contrast to a study conducted in Quetta [8,24]. Food that is too hot or too cold is believed to disturb the menstrual flow and ovarian function and cause acne. A clinical trial indicated that adherence to a ‘snack dietary pattern’ that is a diet rich in foods with a high glycemic index such as desserts, tea, coffee and fruit juices can increase the risk of moderate to severe dysmenorrhea [28]. Hence, healthcare workers should allay any other unnecessary restrictions that are not based on any scientific grounds and recommend a diet rich in iron during menstruation.

The majority of women from the general population experienced fatigue, whereas most women from healthcare workers reported mood swings. In concordance with our results, back pain and pain in the abdomen were the most commonly reported symptoms in surveys conducted in Quetta, Karachi, and Mumbai [8,24,29]. Some of our participants (19.9% of the general population and 18.6% of the healthcare workers) complained of pain during menstruation which deterred them from going out. Menses leave policy has been implemented in the UK, India, and Australia where menstruators are exempted from working while they are experiencing severe pain or discomfort [30]. This can empower the women as it is the acknowledgement of their physiological process. Such policies can be also introduced in Pakistan to facilitate the healthcare workers and general working force.

It is worth mentioning that a comparatively higher number of women from the general population (5.7%) avoided going out of their homes because they feared evil spirits/black magic. A review article highlighted that freedom to participate in daily activities during menstruation empowered women and proved to be an insignia of proper menstrual practice [4]. Fortunately, as per our study, the condition in Pakistan is still better than other Asian countries. For instance, in Nepal, a very strict ritual seclusion of “Chaupaudi” is still practiced where women are ostracized during the entire period of menstruation [4]. Moreover, in India, women are excluded from religious gatherings, and in rural areas, women are restricted from even entering the kitchen [27]. The superstition of the association of menstruation with evil spirits is particularly prevalent in Asia. A menstruating woman deemed impure is more vulnerable to get possessed by demons and hence some women bury the cloths used during menstruation [27]. Even in our study, a few participants from the general population buried or burnt the cloth. Hence, it is the responsibility of healthcare workers to dispel such traditional and cultural myths so that they are not bequeathed to subsequent generations.

In our study, 38.4% of healthcare workers and 31.8% of the general population complained of sanitary pads being unavailable in their institute/college/workplace. A systematic review was conducted to assess the effectiveness of "hardware intervention", that is, the provision of absorbing materials to address the material deprivations and access to WASH facilities [7]. A moderate non-significant effect was observed when reusable homemade and disposable sanitary pads were provided. To assess the true effectiveness of hardware intervention, larger trials should be conducted. Nevertheless, it is still believed that the institutional availability of pads can benefit young girls hailing from a low socio-economic background, as a study in Ghana revealed that school attendance rose by 9% after five months with the provision of disposable sanitary pads [7].

It is important to note that since our targeted population was from only one city, Karachi, it should not be considered an accurate predictor of knowledge, attitude, and practice regarding the menstrual hygiene of the female population of the entire country. Secondly, our study had a narrow coverage of socioeconomic classes, with most participants belonging to the lower and middle class. More versatile studies should be conducted in the future involving a bigger sample size, in order to assess the practices of women of a wider geographical area. The questionnaire was self-designed and many commonly believed myths might have been overlooked. The sample population was not randomized and there may have been a recall bias in answering some questions in the survey.

## Conclusions

Our study found insufficient knowledge about menstruation among the females of Karachi, in both the general population and healthcare workers. Being the principal source of information, mothers with poor knowledge have the potential to propagate and endorse unhygienic practices. Unless we bridge the knowledge gap and destigmatize menstruation, we cannot expect proper menstrual hygiene management by young girls at the time of menarche. To address this void of knowledge and to eliminate incorrect knowledge and subsequent practices, the most efficient approach would be to deal with the problem at the grassroots: by introducing basic reproductive health classes at the middle school level, so that the young girls can have a safe menstrual experience at menarche. Even health care workers harbored some misconceptions, owing to separate compartmentalization of science from religion and culture. It is imperative to rationally reassess and obliterate these myths instead of making them a cultural legacy, passed down to the subsequent generations.

Furthermore, a two-pronged approach is necessary to have a significant impact and for the translation of correct knowledge into correct practices. Therefore, the availability of absorbents and WASH facilities at workplaces and educational institutes is essential to facilitate women and ensure adequate provision of this basic need.

## Appendices

Consent form

The study objective has been explained to me by the researcher and I have understood the information regarding the research project.

I also understand that my participation in this study is entirely voluntary and I voluntarily give my consent to participate in this study.

Name: ____________ (optional)

Signature: _______________

Please note: All responses provided will remain completely anonymous and confidential. The information provided will be solely used for research purposes only. By filling this form, you voluntary give consent to be a part of the research “Perceptions, Practices, and Challenges Regarding Menstrual Hygiene Among Women in Karachi, Pakistan: A Comparison Between General Population and Healthcare Workers”

Socio-demographic profile

1.      Age: ­ _____ years

2.      Education level:

o   Uneducated

o   Primary (Grade 1 to 5)

o   Secondary (Grade 6 to 10/Matric/ O-levels)

o   Intermediate (FSC/FA/A-levels)/Diploma

o   Graduate

o   Postgraduate

3.      Education of mother:

o   Uneducated

o   Primary (Grade 1 to 5)

o   Secondary (Grade 6 to 10/Matric/ O-levels)

o   Intermediate (FSC/FA/A-levels)/Diploma

o   Graduate

o   Postgraduate

4.      Total family income per month:

o   Above PKR 100,000 (1 lac)

o   Between PKR 40,000-100,000

o   Below PKR 40,000

5.      House Structure:

o   Pakka (brick built)

o   Semi Pakka (tin roofing or sheets)

o   Kaccha (no bricks)

6.      Marital Status:

o   Single

o   Married

o   Divorced

o   Widowed

7.      Religion:

o   Islam

o   Christianity

o   Hinduism

Knowledge of the participants regarding menstruation

1.      What was your source of general overview and do’s and don'ts of menstruation?

o   Mother

o   Grandmother

o   Teachers

o   Siblings or relatives

o   Friends

o   Self-knowledge (TV, Books, Internet)

o   No-one

2.      What was your level of knowledge at the time of menarche?

o   I was already aware of periods, knew how to prevent staining my clothes and knew how to properly place the cloth/ pad

o   I was already aware of periods, knew how to prevent staining my clothes but had no idea of how to properly place the cloth/ pad

o   I was aware of periods but didn’t know how to prevent staining my clothes

o   I thought I had some disease/ infection

o   I thought I had suffered an injury

o   I thought I was pregnant

o   I was not aware at all and sought elders

3.      Are you aware of what a tampon is?

o   Yes

o   No

4.      Are you aware of what a menstrual cup is?

o   Yes

o   No

5.      Do you believe that infrequent menses is a sign of pregnancy?

o   Yes

o   No

Reactions and practices of the participants pertaining to menstruation

1.       For those who are aware of tampons, what is the reason for not using them?

o   Unaffordable

o   Uncomfortable/ strange to use

o   Unavailability in local shops

o   Affects virginity

2.      Are you able to talk freely with your mother or any relatives about any gynecological issues?

o   Yes

o   No

3.      Do you avoid taking bath during menses?

o   Yes

o   No

4.      If yes, how many days do you avoid bathing during menses?

o   During the first day

o   During the first three days

o   During all days

5.      Please tick the reasons for taking or avoiding bath during menses

For those who take bath: 

o   Soothes cramps

o   Hygienic reasons

o   Feel much better after bath

For those who avoid bath: 

o   Causes cramps

o   Causes irregular flow

o   Causes water to fill in stomach

o   Advised by elders not to take one

6.      Do you avoid washing the groin after urination/ defecation?

o   Yes

o   No

7.      Do you stop doing exercise during menses?

o   Yes

o   No

o   I don’t do exercise regularly

8.      Do you have any restrictions of activity during menses?

o   Yes

o   No

9.      If yes, which ones?

o   Climbing stairs

o   Lifting weight

o   Walking

10.      What were your feelings when you menstruated for the first time? (you can mark more than one option)

Scared

o   Yes

o   No

Guilty

o   Yes

o   No

Upset

o   Yes

o   No

Anxious

o   Yes

o   No

Normal

o   Yes

o   No

Confused

o   Yes

o   No

Miserable

o   Yes

o   No

Shame

o   Yes

o   No

Excited/Delighted

o   Yes

o   No

11.      Which of these do you use during menstruation to avoid staining your clothes?

o   Cloth

o   Pads

o   Cloth and pads

o   Tampon

o   Tissue paper

o   Cotton

o   Menstrual cup

12.      For those using cloth, is the cloth:

o   Torn from a new fabric

o   Torn from an old worn out shirt

13.      How many times do you change the menstrual product?

o   4 or more times per day

o   2-3 times per day

o   Once per day

o   Use 1 for almost 2 days

14.      How do you dispose of the menstrual products after using them?

o   Bury it

o   Burn it

o   Flush it in the toilet

o   Throw it in waste

o   Throw it in sea

o   Store it for further use

15.      What do you use while taking a bath after your period ended?

o   Water only

o   Water and soap

o   Water and antiseptic

Problems and challenges faced by participants during menstruation

1.      Do you abstain from eating certain food items during menses?

o   Yes

o   No

2.      If yes, which of these food items do you abstain from: (you can mark more than one option)

o   Guava

o   Papaya

o   Oily food

o   Pickle

o   Meat

o   Spicy food

o   Curd/ Yoghurt

o   Caffeine

o   Egg

o   Milk

o   Fish

o   Cold drink/ Cold water/ Ice cream

3.      Which one of these complains do you have during menses? (you can mark more than one option)

Tension or anxiety

o   Yes

o   No

Poor concentration

o   Yes

o   No

Abdominal bloating

o   Yes

o   No

Change in sexual desires

o   Yes

o   No

Breast tenderness

o   Yes

o   No

Crying spells

o   Yes

o   No

Hair fall or dandruff

o   Yes

o   No

Acne

o   Yes

o   No

Mood swings or irritability

o   Yes

o   No

Joint or muscle pain

o   Yes

o   No

Constipation or diarrhea

o   Yes

o   No

Appetite changes or food cravings

o   Yes

o   No

Headache

o   Yes

o   No

Nausea or vomiting

o   Yes

o   No

Trouble falling asleep

o   Yes

o   No

Fatigue

o   Yes

o   No

Abdominal pain

o   Yes

o   No

Social withdrawal

o   Yes

o   No

4.      If you experience abdominal pain during menses, what do you do to relieve it?

o   Self-medication

o   Consulting a gynecologist

o   Herbs/ Homeopathic medicines

o   Homemade foods: turmeric milk, tea, hot porridge, soup, hot fluids

o   Sleep

o   Do nothing

5.      Do you avoid taking any medicine during menses? (fear that the menses will stop)

o   Yes

o   No

6.      Regarding going out of your home during periods:

o   I avoid going out because I experience a lot of pain

o   I avoid going out because I feel my energy gets low during periods

o   I avoid going out because I’m scared of the evil spirits/ black magic

o   I avoid going out because I’m scared of staining my clothes accidentally

o   I go out of my home without any inhibitions

7.      Do you feel that you have a bad odor during periods?

o   Yes

o   No

8.      What is the reason for using cloth/ tissue paper/ cotton?

o    Pads are uncomfortable to use

o    Pads are unavailable in nearby shops

o    Pads are unaffordable

o    I feel shy to buy pads because the shopkeepers are male

o    I feel shy asking my family men to buy pads for me

9.      Have you ever had? (you can mark more than one option)

Any reproductive tract infection

o   Yes

o   No

Foul smelling/ abnormal discharge

o   Yes

o   No

Painful sores on pubic area

o   Yes

o   No

Rashes on pubic area

o   Yes

o   No

Pain or cramps during periods

o   Yes

o   No

Early period

o   Yes

o   No

Delayed period

o   Yes

o   No

Missed/ infrequent period

o   Yes

o   No

Light flow

o   Yes

o   No

Heavy flow

o   Yes

o   No

No issues

o   Yes

o   No

10.     Did you seek any treatment for these gynecological issues?

o   Yes

o   No

11.      Are there sanitary pads available in your institute/ college/ workplace?

o   Yes

o   No

o   I stay at home
